# An interlaboratory proficiency test using metagenomic sequencing as a diagnostic tool for the detection of RNA viruses in swine fecal material

**DOI:** 10.1128/spectrum.04208-23

**Published:** 2024-08-20

**Authors:** Lihong Liu, Mikhayil Hakhverdyan, Per Wallgren, Kevin Vanneste, Qiang Fu, Pierrick Lucas, Yannick Blanchard, Miranda de Graaf, Bas B. Oude Munnink, Sander van Boheemen, Alex Bossers, Marcel Hulst, Steven Van Borm

**Affiliations:** 1 Department of Microbiology, Swedish Veterinary Agency, Uppsala, Sweden; 2 Department of Animal Health and Antimicrobial Strategies, Swedish Veterinary Agency, Uppsala, Sweden; 3 Department of Transversal activities in Applied Genomics, Sciensano, Brussels, Belgium; 4 Ploufragan-Plouzané-Niort Laboratory, French Agency for Food, Environmental and Occupational Health Safety, Ploufragan, France; 5 Department of Viroscience, Erasmus University Medical Center, Rotterdam, the Netherlands; 6 Department of Epidemiology, Bioinformatics and Animal models, Wageningen BioVeterinary Research, Wageningen University & Research, Lelystad, the Netherlands; 7 Department of Avian Virology and Immunology, Sciensano, Ukkel, Belgium; Ross University School of Veterinary Medicine, Basseterre, Saint Kitts and Nevis

**Keywords:** diagnostics, metagenomics, porcine astrovirus, proficiency testing

## Abstract

Metagenomic shotgun sequencing (mNGS) can serve as a generic molecular diagnostic tool. An mNGS proficiency test (PT) was performed in six European veterinary and public health laboratories to detect porcine astroviruses in fecal material and the extracted RNA. While different mNGS workflows for the generation of mNGS data were used in the different laboratories, the bioinformatic analysis was standardized using a metagenomic read classifier as well as read mapping to selected astroviral reference genomes to assess the semiquantitative representation of astrovirus species mixtures. All participants successfully identified and classified most of the viral reads to the two dominant species. The normalized read counts obtained by aligning reads to astrovirus reference genomes by Bowtie2 were in line with Kraken read classification counts. Moreover, participants performed well in terms of repeatability when the fecal sample was tested in duplicate. However, the normalized read counts per detected astrovirus species differed substantially between participants, which was related to the different laboratory methods used for data generation. Further modeling of the mNGS data indicated the importance of selecting appropriate reference data for mNGS read classification. As virus- or sample-specific biases may apply, caution is needed when extrapolating this swine feces-based PT for the detection of other RNA viruses or using different sample types. The suitability of experimental design to a given pathogen/sample matrix combination, quality assurance, interpretation, and follow-up investigation remain critical factors for the diagnostic interpretation of mNGS results.

**IMPORTANCE:**

Metagenomic shotgun sequencing (mNGS) is a generic molecular diagnostic method, involving laboratory preparation of samples, sequencing, bioinformatic analysis of millions of short sequences, and interpretation of the results. In this paper, we investigated the performance of mNGS on the detection of porcine astroviruses, a model for RNA viruses in a pig fecal material, among six European veterinary and public health laboratories. We showed that different methods for data generation affect mNGS performance among participants and that the selection of reference genomes is crucial for read classification. Follow-up investigation remains a critical factor for the diagnostic interpretation of mNGS results. The paper contributes to potential improvements of mNGS as a diagnostic tool in clinical settings.

## INTRODUCTION

Untargeted metagenomic methods rely on high-throughput sequencing and bioinformatic analysis of sequencing data to identify microbes present in samples without prior targeted cultivation or amplification of genomes (1). This approach, referred to below as metagenomic shotgun sequencing (mNGS), is extensively used to study microbiota in different fields. As a generic method, it has become the standard for the detection of unknown, emerging, or re-emerging pathogens affecting humans, animals, or plants (2). It provides a considerable benefit for the investigation of emerging animal and public health threats. For example, this approach allowed the identification of a novel orthobunyavirus (Schmallenberg virus) in dairy cattle exhibiting fever, reproductive disorders, decreased milk production, and diarrhea (3). Subsequent real-time RT-PCR screening, as well as the experimental inoculation of infectious blood into calves, confirmed that the virus was responsible for the previously unidentified disease. Occasional animal-to-human transmission of pathogens could trigger serious public health crises, as exemplified by the ongoing global pandemic of coronavirus disease 2019. The causative agent, severe acute respiratory syndrome coronavirus 2, was identified by metagenomic sequencing of bronchoalveolar lavage fluid from patients in China (4, 5) and confirmed by virus isolation and transmission electron microscopy (5). Both examples highlight the power of mNGS as a diagnostic tool.

The major steps of mNGS comprise sample disintegration, nucleic acid extraction, cDNA and second-strand synthesis in case of RNA, fragmentation, library preparation and sequencing, and bioinformatic data analysis (6). Minor additional steps to the overall scheme of the metagenomic protocol can significantly improve the results. For example, deep-frozen-based sample disintegration methods are generally able to maintain high-quality nucleic acids, resulting in an overall good performance of mNGS (7). While PCR amplification of DNA libraries is generally performed to increase the signal strength in metagenomic diagnostics, a workflow without this additional amplification step allows an unbiased pathogen detection in a variety of sample matrices, including pig feces (7). A longer homogenization time and size-selective filtration can help improve the enrichment of viral particles (8). A modular approach using enrichment and amplification steps claimed minimal bias on the detected virome as demonstrated on mock communities of relevant reference (9) while allowing workflow choices suited, e.g., challenging sample matrices (10). The hybridization capture enrichment approach also shows a high diagnostic performance (11–13). Although some studies have highlighted the value of pre-treatment or amplification steps for certain combinations of pathogens, the sample type, and the required analytical sensitivities (9, 14), the mNGS user should be fully aware of methodological biases as previously documented. For instance, certain filtration steps may remove large viruses (9, 15), while random amplification methods may over-amplify certain genomic regions (15) or small circular genomes and under-amplify genomes with extreme GC content (16). Sequencing library preparation methods have the potential to induce bias in the detected virome (17). Lastly, bioinformatic methods may induce biases in classifying mNGS reads (18). These potential methodological biases complicate the diagnostic validation of mNGS as a catch-all generic diagnostic assay and highlight the importance of cautious interpretation and follow-up of mNGS findings.

Although metagenomic detection of pathogens has been applied in both human and veterinary laboratories, hurdles like the presence of largely unknown quantities of other non-relevant nucleic acids are still present for its routine application as a diagnostic method (19, 20). The methodology has not been routinely evaluated by proficiency testing (PT) of the same set of clinical samples in multiple laboratories, whereas this is routinely the case for targeted nucleic acid detection assays. The use of PT as an external assessment tool is essential to evaluate and verify mNGS quality and reliability (21). Previous PT evaluations have focused on the reproducibility of data analysis steps using simulated mNGS data sets (21), samples spiked with a mock microbial community (22), or mock communities (23). A Swiss PT between clinical mNGS laboratories evaluated both clinical samples spiked with known viruses, as well as a distributed mNGS data set to disentangle variability arising from the laboratory compared to the bioinformatic parts of the workflow (24, 25).

The astrovirus genome is a single-stranded positive-sense RNA molecule of approximately 7 kb in size and contains three overlapping open reading frames and a poly-A tail. The virus is found in a wide range of hosts such as humans, birds, and mammals. Porcine astroviruses (PAstVs) belong to the *Mamastrovirus* genus of the *Astroviridae* family. While porcine astrovirus 1 (PAstV1) is listed as the only officially approved species, additional PAstVs that are distinct phylogenetically from PAstV1, e.g., PAstV2, PAstV3, PAstV4, PAstV5, and mamastrovirus 3 (MAstV3), remain unclassified but are continuously reported in the literature. The presence of all five species in a single pig farm in the USA has been documented (26), and co-circulation of more than one species has been reported in 15 out of the 17 investigated pig farms (27).

The main goal of the present study was to evaluate the suitability of selected mNGS workflows to identify RNA viruses using a well-characterized pig fecal material natively containing multiple PAstVs. We also aimed to evaluate repeatability in identifying PAstVs in the fecal samples that were tested in duplicate.

## MATERIALS AND METHODS

### Organization of the proficiency test

The viral metagenomic PT was organized within the One Health European Joint Programme (European Union’s Horizon 2020 Research and Innovation programme under grant agreement No 773830) internal joint research project METASTAVA (Standardisation and validation of metagenomic methods for the detection of foodborne zoonoses, antimicrobial resistance and emerging threats). The following six European public and animal health institutes from five countries (shown in alphabetical order) participated in the PT: ANSES (France), ErasmusMC, (EMC, the Netherlands), Friedrich-Loeffler-Institut (FLI, Germany), Sciensano (Belgium), Swedish Veterinary Agency (SVA, Sweden, the organizer of the PT), and Wageningen BioVeterinary Research (WBVR, the Netherlands). Six partners registered their interest in participating in this PT and agreed to carry out sequencing according to the specified workflows and procedures (Table 1) and subsequent standardized data analysis (File S1) with the implementation of a mapping quality cut-off criterion. A native sample was used rather than a mock community spiked with defined microbes in the PT to reflect the complexity of sample matrices handled daily by the participants. An astrovirus-positive fecal material from a healthy pig was selected since astroviruses are non-zoonotic RNA viruses that could be handled in biosafety level 2 laboratories by all participating institutes. Thereafter, the organizer identified and evaluated a suitable sample for the PT, thoroughly characterized the material, prepared 250 mg fecal sample aliquots and RNA extracts, and sent these materials on dry ice to each participant. Participants also received the same reference database to be used for Kraken taxonomic analysis (28) and the reference genomes of astroviruses for Bowtie2 alignment (29) of sequencing reads. Participants performed sample processing, sequencing, and sequence analysis and reported files including raw data, quality reports, Kraken mapping results, Bowtie2 alignment results, numbers of reads for porcine astrovirus species following the conventional nomenclature, and a short report of the methods and analysis. An additional centralized analysis of all PT data from the six participants was performed, and additional sequences of PAstVs were retrieved from NCBI as references that were not distributed to the participants.

**TABLE 1:** Modules from the METASTAVA mNGS workflow applied by the participants

Participant	Sample preparation			dsDNA synthesis		Library preparation		Sequencing	
	Disintegration	Extraction	DNase	Reagents	Elution	Kits	#cycles	Platform	Chemistry
P1	Vortexing	TRIzol + RNeasy Kit	No	SuperScript IV + NEBNext	20 µL	Nextera XT	12	NovaSeq/6000	NovaSeq 6000 SP Reagent Kit
P2	Bead-beating (FastPrep)	High Pure RNA Isolation Kit	No	SuperScript IV + NEBNext	10 µL	Nextera XT	20	MiSeq	MiSeq Reagent Kit v3
P3	CryoPREP	TRIzol + RNAadvance Kit	Yes	SuperScript IV + NEBNext	25 µL	Covaris AFA + Gene Read L Core	0	Ion Torrent S5XL	Ion Xpress
P4	Bead-beating (Tissuelyser) (4°C pre-cooled)	TRIzol + RNeasy Kit	No	SuperScript IV + NEBNext	15 µL	Nextera XT	12	MiSeq	MiSeq Reagent Kit v3
P5	Bead-beating (FastPrep)	TRIzol + RNeasy Kit	Yes	SuperScript IV + NEBNext	20 µL	Nextera XT	12	MiSeq	MiSeq Reagent Kit v3
P6	Bead-beating (FastPrep)	High Pure RNA Isolation Kit	Yes	SuperScript IV + NEBNext	30 µL	Nextera XT	12	MiSeq	MiSeq Reagent Kit v3

### Characterization of the PT sample

The pig fecal material was obtained from a 12-week-old healthy pig in a specific pathogen-free farm in Sweden. The material tested positive for porcine astrovirus by real-time RT-PCR (30). RNA was extracted from seven aliquots of the fecal material by a combined TRIzol reagent (ThermoFisher Scientific, Carlsbad, CA) and RNeasy Mini kit (Qiagen, Hilden, Germany), eluted in 40 µL nuclease-free water, mixed, and aliquoted (40 µL final volume). Both fecal and RNA aliquots were stored at −80°C until further testing. The presence of PAstVs in the sample was confirmed by preliminary mNGS analysis prior to the PT. In addition, the viral load was quantified as 5.5 × 10^5^ copies/mg for PAstV2 with primers/probe PAstV2-F/P/R, 9.8 × 10^4^ copies/mg for PAstV4 with PAstV4-F/P/R, 2.4 × 10^3^ copies/mg for mammalian astrovirus 3 (MAstV3) with MAstV3-F/P/R, and 5.6 × 10^1^ copies/mg for PAstV5 with PAstV5-F/P/R and the absence of PAstV3 with PAstV3-F/P/R by five species-specific real-time RT-PCR assays using synthetic DNA standards for quantification. The list of nucleotide sequences is provided in Table S1. The full description of MiSeq sequencing, astrovirus sequence alignment and phylogeny reconstruction, real-time RT-PCR assays, and validation of their specificity is presented in File S2.

### mNGS sequencing

The participants used various methods for sample disintegration and nucleic acid extraction from the fecal material, cDNA synthesis and second-strand synthesis, fragmentation, library preparation, and high-throughput sequencing (Table 1). The participants aimed for at least 4 million reads per sample.

### Metagenomic read classification

A standardized prescribed data analysis procedure (File S1) was followed by all participants to rule out potential variation introduced by using different bioinformatic approaches, using Kraken for k-mer-based taxonomic classification against a provided version of the NCBI RefSeq Microbial Genomes database (28), which lists both classified and unclassified PAstVs as “species.” In addition, read mapping using Bowtie2 (29) against a list of reference genomes provided to the participants was used for the semiquantitative species-level quantification of different astroviruses in comparison to the species classification provided by Kraken. For Bowtie2 read alignment data analysis, the participants received a multi-FASTA file containing reference genomes for eight porcine astrovirus species and one dromedary astrovirus that is closely related to PAstVs (Fig. S1), for which accession numbers are provided in Table 2 and pairwise sequence identity is presented in Table S2.

**TABLE 2:** Porcine astrovirus reference sequences used in this study

Species	NCBI reference genome	Strain name
Porcine astrovirus 4^*a*^	NC_023675.1	Porcine astrovirus 4 strain 35/USA
(PAstV4)	NC_016896.1	Astrovirus wild boar/WBAstV-1/2011/HUN
Porcine astrovirus 2^*a*^	NC_023674.1	Porcine astrovirus 2 strain 43/USA
(PAstV2)	NC_027711.1	Dromedary astrovirus isolate DcAstV-274
	NC_034974.1	Mamastrovirus 2 isolate K321
Porcine astrovirus 3 (PAstV3)	NC_019494.1	Porcine astrovirus 3 isolate US-MO123
Porcine astrovirus 5 (PAstV5)	NC_023636.1	Porcine astrovirus 5 isolate AstV5-US-IA122
Mamastrovirus 3 (MAstV3)	NC_025379.1	Mamastrovirus 3 isolate PAstV-GX1
Bastrovirus	NC_032423.1	Bastrovirus/Vietnam/porcine/17489_85

^*a*^ PAstV4 and PAstv2 are considered as cluster species consisting of two and three closely related astrovirus species, respectively.

### Data integration and final evaluation

Result files from all participants were processed centrally at P5 using the R package tidyverse (version 1.3.1) and R (version 4.1.2) on a Windows platform [x86_64-w64-mingw32/x64 (64-bit)] in the environment RStudio Desktop (version 2021.09.2). Basic statistics of reads (numbers and lengths) were obtained by aggregating FastQC (31) results of all participants into a single file. For the processing of the Kraken results, the numbers of reads for *Astroviridae* family and porcine astrovirus species, as well as the total number of reads, were extracted directly from the Kraken reports provided by the participants. For the processing of Bowtie2 results, read counts mapping to reference genomes of porcine astrovirus species as reported by the participants themselves were used. For both Kraken and Bowtie2 results, the number of viral reads “on-target” was normalized as reads per million (RPM), and we used RPM, unless explicitly stated otherwise in this report. The coefficient of variation (CV) for *Astroviridae* RPM was calculated by dividing the standard deviation of RPM by the average RPM.

Apart from summarizing the reported participants’ results, the organizer performed a central Bowtie2 mapping of the reads from all participants’ data sets following the same bioinformatic pipeline at the Uppsala Multidisciplinary Center for Advanced Computational Science, a CentOS (version 7) Linux platform. The Illumina paired-end reads were trimmed prior to mapping to the reference sequences according to the pipeline (File S1) whereas no trimming was done for the Ion Torrent single-end reads. The reads from all participants were mapped to 985 sequences of PAstVs that were retrieved from GenBank by filtering out species of non-porcine origin followed by manual examination. Those mapped reads were further assembled into contigs by SPAdes (version 3.15) using the default settings (32). The species of each contig was determined by either blastn analysis against and/or phylogenetic relationship with the porcine astrovirus sequences of known species. The accession numbers of porcine astrovirus sequences and corresponding species are presented in Table S3. Finally, the species of the mapped reads were determined by Bowtie2 mapping to the contigs.

The sequencing files and metadata were deposited in the European Nucleotide Archive under project number PRJEB44508.

## RESULTS AND DISCUSSION

### Metagenomic data produced by the participants

All participants successfully completed sample preparation, sequencing, and sequence analysis. The number of reads and libraries and read length are shown in Table 3. All participants tested the fecal material in duplicate, except P2 who tested one fecal and one RNA in two separate sequencing experiments using the same libraries. Although testing the RNA sample in duplicate was not required, two participants (P1 and P5) did the test in duplicate. P3 using the Ion Torrent S5XL platform generated, on average, 2.1 million reads per library, while participants P2, P4, P5, and P6 using the Illumina MiSeq produced on average 4.9 million reads, ranging from 3.0 to 6.4 million per sample. P1 used the Illumina NovaSeq platform and produced the largest number of reads (9.4 million) per sample. Read lengths also varied but mainly depended on the sequencing chemistry and platform.

**TABLE 3:** Basic statistics of metagenomic PT raw data

Participant	Mean # reads per sample (million)	# libraries	Read length
P1	9.4	4	151
P2	3.0	2	300
P3	2.1	5	25–615
P4	4.8	3	35–301
P5	6.4	4	35–301
P6	5.5	3	35–151

### Identification of sequences representing the family *Astroviridae*

**Fig 1:**
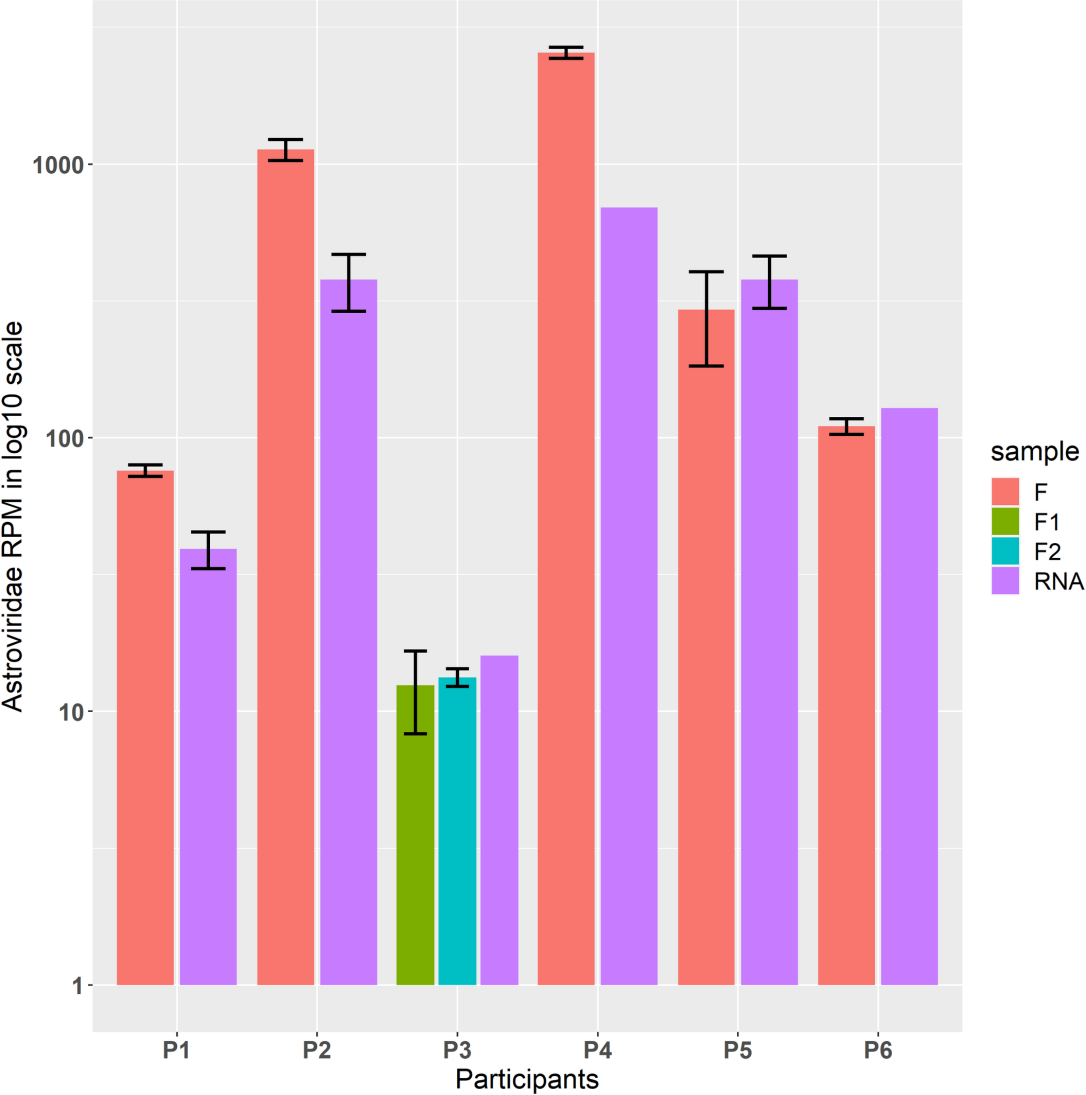
The average RPM obtained using Kraken. The error flags represent the standard deviation for all the samples except for three RNA samples that were tested only once by P3, P4, and P6. P3 tested each fecal sample fecal_1RNA and fecal_2RNA in duplicate.

All participants identified *Astroviridae* by k-mer taxonomic classification (Kraken) against the provided database using the same bioinformatic workflow (File S1). A summary of average RPM values for the detection of astroviruses at the family level is shown in Fig. 1. Of the six participants, P4 reported the highest *Astroviridae* RPM in both the fecal material (2,552) and the RNA sample (693), which was followed by P2 with an RPM of 1,130 in the fecal material and 378 in the RNA sample. Both P2 and P4 applied the same methods for cDNA synthesis, library preparation, and sequencing on an Illumina MiSeq platform as used by P5 and P6 (Table 1). Only P4 reported a pre-cooling at 4°C of all buffers and components (including aluminum sample blocks) prior to bead-beating sample homogenization (33), likely resulting in an improved RNA quality and consequent sequencing data generation. P3 produced a low RPM value in both fecal samples (average 13 RPM) and RNA (16) based on the Kraken analysis. P3 employed cryoPREP disintegration, which may have resulted in different host/bacterial/viral nucleic acid ratios compared to bead-beating methods. Another difference of the P3 workflow is the absence of an amplification step during sample processing, which can bias the ratio of different taxa of the sample community in the sequencing data set.

While three participants (P3, P5, and P6) identified a similar level of astrovirus RPM in both the fecal material and the RNA, the other three participants (P1, P2, and P4) reported a substantially higher astrovirus RPM in the fecal material than in the RNA (Fig. 1). The only module (Table 1) differentiating both groups was DNase treatment, which was co-incidentally employed by participants P3, P5, and P6. Since the original RNA had been treated with DNase via on-column digestion during extraction, treatment of the fecal material resulted in a similar level of astrovirus RPM in both RNA and fecal material. On the other hand, participants P1, P2, and P4 reported higher levels of astrovirus RPM in the fecal material than in the original DNase-treated RNA, suggesting that the DNase treatment of the fecal material might have caused unexpected side effects, which requires a full investigation. It is likely that the effects of DNase treatment on the enrichment of viral RNA may depend on the matrices. Apart from the DNase treatment, there were some overlaps of the modules that might also have caused the differences. Enrichment by filtration and nucleases (both DNase and RNase) increased virus reads in nasal swabs and lungs spiked in with six viruses, but not the feces spiked with the same viruses (34).

### Repeatability of *Astroviridae*-classified mNGS reads

The CV within participating laboratories for *Astroviridae* RPM obtained by Kraken was 4.8% (P1), 8.8% (P2), 33.5% for fecal_1 and 7.4% for fecal_2 (P3), 4.6% (P4), 37.8% (P5), and 6.6% (P6), indicating an overall good repeatability of the metagenomic identification of *Astroviridae* in duplicate testing of the porcine fecal material. Except for P5, which had the highest CV, the overall repeatability within laboratories was high (where sufficient data were available; also see Fig. 1). The variation was larger between laboratories. Large differences in reads assigned to microorganisms were also reported in a PT of smoked salmon spiked with a mock community (22). For example, data sets M33 and M34 were generated following the same workflow, including filtration and endonuclease treatment, yet they differed by 3-fold in the percentage of host (Eukaryota), 0.5-fold in the percentage of bacteria, and 12.7-fold in the percentage of viruses. Due to the different combinations of the mNGS laboratory methodological modules (Table 1) that were applied by various participants, substantial variations among laboratories were anticipated. These results underscore the significance of meticulous design and validation of mNGS workflows. Another related study emphasized the necessity of several technical steps to conduct a successful interlaboratory study using high-throughput sequencing methods or mNGS (23), and differences between methods could substantially impact the results (35).

### Species-level astrovirus detection by Kraken and Bowtie2

Metagenomic identification of astroviruses in the porcine fecal material at the species level was also evaluated. All participants correctly identified the two most abundant porcine astrovirus species PAstV2 and PAstV4 in the fecal and RNA samples with Kraken (Table 4: left panel) and Bowtie2 (Table 4: right panel). The corresponding viral load was 5.5 × 10^5^ copies/mg (PAstV2) and 9.8 × 10^4^ copies/mg (PAstV4). RPM values at the species level varied considerably among participants. Furthermore, all participants (except P3) reported low RPM values of MAstV3 (corresponding to 2.4 × 10^3^ copies/mg) in the fecal and/or RNA samples. P4 and P6 identified the least abundant species PAstV5 (corresponding to 5.6 × 10^1^ copies/mg) in the fecal material using Bowtie2. P4 reported PAstV3 in the fecal material, whereas P6 reported PAstV3 in the RNA sample using Bowtie2. This likely constitutes a false positive result as the presence of this species was not confirmed by the PAstV3-specific real-time RT-PCR assay. Indeed, P4 found that the few reads that had been aligned to PAstV3 failed to form a contig, and a close investigation of the reads discovered the presence of a poly-A/T stretch in the sequences that adversely affected the quality of Bowtie2 alignment. Therefore, the genome location to which the reads are mapped should also be taken into consideration when evaluating and reviewing mNGS results, besides the depth of coverage and number of different genome regions covered by the reads (36). As highlighted by the differences in the normalized *Astroviridae* reads (Fig. 1), methodological differences in the workflow clearly have an impact on the detection sensitivity of low-abundant species. Moreover, this PT did not consider a significance cut-off for metagenomic findings. The study (33) suggests an arbitrary significance criterion of RPM >1, which in the context of the present PT would question the significance of most (except P2 and P4) MAstV3 and all PAstV5 identifications based on low RPM. However, such a cut-off or threshold would have to be determined for every protocol and validated by comparing with golden standard molecular techniques, as recommended (36). It is strongly advised to initiate follow-up investigations to confirm the presence of low RPM taxa.

**TABLE 4:** Analytic performance of metagenomic identification of porcine astrovirus species in both fecal (F) and RNA (R) samples by the six participants^*a*^

Participant	Type	RPM (Kraken)				RPM (Bowtie2)				
		PAstV2 (5.5 × 10^5^)	PAstV4 (9.8 × 10^4^)	MAstV3 (2.4 × 10^3^)	PAstV5 (5.6 × 10^1^)	PAstV2 (5.5 × 10^5^)	PAstV4 (9.8 × 10^4^)	MAstV3 (2.4 × 10^3^)	PAstV5 (5.6 × 10^1^)	PAstV3 (−)
P1	F	21	45.7	0.1	−	37.2	46.1	0.9	−	−
	RNA	10.1	23.9	−	−	18.2	27	0.3	−	−
P2	F	297.4	602.6	1.2	−	303.6	349.4	6.3	−	−
	RNA	122.5	189.9	2.4	−	122.1	113.9	5.9	−	−
P3	F1	3	7.3	−	−	7.2	10	−	−	−
	F2	3.9	7.3	−	−	9.2	11.2	−	−	−
	RNA	6	7.8	−	−	11.7	11.2	−	−	−
P4	F	828	1,270.6	3.3	0.3	721.9	768.2	4.9	0.5	0.2
	RNA	208.8	373.5	0.8	−	191.6	237.5	1.1	−	−
P5	F	83.7	164	0.2	−	80.2	99.1	1	−	−
	RNA	120.5	199.3	0.3	−	121.3	117.8	0.6	−	−
P6	F	30.1	63.1	0.3	−	26.7	40.4	0.4	0.2	−
	RNA	41.8	62.3	0.4	−	38.5	42	0.4	−	0.2

^*a*^ Numbers refer to the average RPM per sample obtained by using Kraken (left panel) or Bowtie2 (right panel). A dash sign indicates a non-detection of the category.

In the PT, all participants were successful in sample preparation, sequencing, and data analysis, indicating that the selected workflows were rather robust. P3 interpreted and reported only the numbers of reads for the porcine astrovirus species, fulfilling the objective of this PT, whereas the remaining five participants reported the numbers of reads for all astrovirus species without differentiation. As pointed out in another PT (37), it is necessary to build the capacity for the interpretation of diagnostic metagenomic data sets, and in the context of this PT, it is probably the lack of awareness that led the five participants of this PT not to report the results as required.

### Investigation of the effect of selected reference sequences on Bowtie2 read alignment

Although all participants reported the presence of the two dominant porcine astrovirus species in the samples, the RPM values for all detected species were inconsistent with the viral loads as quantified by real-time RT-PCR assays. This raised a concern about the suitability of the software and references used in this PT data analysis. An independent centralized Bowtie2 mapping of the PT data reported by all participants to the nine reference genomes (Table 2) identified more reads (not RPM) for PAstV4 (13,442 reads) than PAstV2 (12,257 reads) and the presence of MAstV3 (124 reads), PAstV5 (5 reads), and PAstV3 (2 reads) in the materials. The pattern of the identified porcine astrovirus species composition was similar to those reported by the participants, excluding the computational environment, software, and analysis operator of each participant as the main factors causing the observed discrepancies between real-time RT-PCR quantification and Bowtie2 mapping results. Thereafter, additional Bowtie2 analyses without a MAPQ cut-off criterion were made focusing on the used reference genomes for mNGS read classification.

Reads from the pre-PT MiSeq characterization were used to assemble contigs by CLC Genomics Workbench version 11.0.2 (CLC Bio-Qiagen), and the corresponding species of the 21 contigs over 500 nt long were determined by blastn analysis against 1,094 sequences of PAstVs. Of the 21 contigs, 11 were PAstV4, 9 were PAstV2, and 1 was MAstV3. Bowtie2 mapping of all reads from the six participants to the 21 contigs determined 151,900 astrovirus reads, including 99,893 (65.8%) reads of PAstV2, 51,790 (34.1%) reads of PAstV4, and 9 (0.01%) reads of MAstV3. The number of the reads for each species or the relative abundance more closely matched the viral load as determined by the real-time RT-PCR quantification, indicating the importance of the selection of reference genomes in mNGS read classification.

The unproportionally lower number of reads for MAstV3 and the absence of PAstV5 in the pre-PT MiSeq data suggested the need for references closely related to the porcine astrovirus species in the material. This would enable Bowtie2 to identify and assign reads to both species. Therefore, a similar approach was taken to prepare the contigs assembled from all reads from the six participants, as references, and the species of the contigs were determined by blastn analysis against 1,007 sequences (Table S3). Out of the 113 million total reads produced by six participants (P2: not in duplicate), 106,289 reads were found to be PAstVs and consisted of 66,271 (62.3%) PAstV2, 39,899 (37.5%) PAstV4, 93 (0.09%) MAstV3, and 26 (0.02%) PAstV5. The composition of overall porcine astrovirus species correlated perfectly with viral load in the material. Thus, for a given Bowtie2 algorithm for the classification of reads, the selection of reference sequences closely related to the species in the sample data (here modeled by the use of *de novo* contigs as references) had a large impact on quantitative performance. Although such prior knowledge about target species in samples is in theory impossible in diagnostic mNGS applications, these modeling efforts show the importance of proper reference data selection for a given classification algorithm on mNGS read classification and warn for cautiously selecting reference databases fit for purpose. The quality of the *de novo* contigs can be assessed and even corrected by new tools such as metaMIC (38). Inclusion of the *de novo* contigs in the regularly updated reference database would be a great strategy for improving mNGS as a generic diagnostic method in both veterinary and public health.

### Conclusions

Results of this study indicated that careful design, validation, and subsequent execution of various stages of the mNGS workflows play a crucial role in determining the final observed outcomes. All six participants of this PT identified PAstVs at the *Astroviridae* family level in the fecal material and the extracted RNA. The normalized number of astrovirus reads varied substantially among participants and sequencing methodologies. Participants performed well in terms of repeatability when the fecal material was tested in duplicate, resulting in a low coefficient of variation. All participants classified the majority of reads to two porcine astrovirus species (PAstV2 and PAstV4) by both approaches. Further centralized analysis indicated the importance of selecting the proper references for mNGS classification. The awareness of result review and reporting needs to be raised, and follow-up investigations are required to verify the presence of viruses with just a few reads.

## SUPPLEMENTAL MATERIAL

Supplemental data 1 spectrum.04208-23-s0001.pdf Pipelines.

Supplemental data 2 spectrum.04208-23-s0002.pdf Characterization.

Fig. S1 spectrum.04208-23-s0003.pdf Phylogeny.

Table S1 spectrum.04208-23-s0004.pdf Nucleotide sequences.

Table S2 spectrum.04208-23-s0005.pdf Pairwise sequence identity.

Table S3 spectrum.04208-23-s0006.pdf Accession numbers.

ASM does not own the copyrights to Supplemental Material that may be linked to, or accessed through, an article. The authors have granted ASM a non-exclusive, world-wide license to publish the Supplemental Material files. Please contact the corresponding author directly for reuse.
