# Supplementary material for: An interlaboratory proficiency test using metagenomic sequencing as a diagnostic tool for the detection of RNA viruses in swine fecal material
Source: Microbiol Spectr. 2024 Aug 20;12(10):e04208-23. doi: 10.1128/spectrum.04208-23 (PMC11448438; doi:10.1128/spectrum.04208-23)
Supplement: Supplemental data 1 — Pipelines. [file spectrum.04208-23-s0001.pdf]

Metastava Analysis pipeline specification v1.1

Date: 2019-03-07
Tags: *metastava*
Created by: Qiang FU

Goal :

This document describes the exact tools, options, and database(s) (where applicable) used to analyze metagenomics data generated within the Metastava project.

Analysis Procedure :

The NGS data of a sample generated by means of the Illumina platform is analyzed by following the sequential steps listed below, with the tools used in parentheses:

- raw reads quality check (FastQC)
- reads poly A/T tail trimming (Prinseq)
- reads quality trimming (Trimmomatic)
- trimmed reads quality check (FastQC)
- reads taxonomy profiling (Kraken)
- taxonomy profile report generation (Kraken)
- optional: taxonomy profile visualization (Krona)
- read mapping against target species (Bowtie2)

The detailed information about each step is described in the next sections. Note that the analysis pipeline expects paired-end NGS sequencing data, with two gzipped Fastq files as input, one containing the sequences of forward reads and one the reverse reads.

Analysis Step Specifications:

In the following subsections, specifications for each step are given with an example of the required command. Here, a dummy example sample **SAMPL1** with gziped fastq files *SAMPL1_L001_R1_001.fastq.gz* and *SAMPL1_L001_R2_001.fastq.gz* containing the raw read sequences and a REFERENCE.fasta file containing reference genome sequences of species of interest are used to demonstrate the analysis procedure.

Metastava Analysis pipeline specification v1.1

Date: 2019-03-07
Tags: *metastava*
Created by: Qiang FU

Raw Reads Quality Check (FastQC)

In this step, FastQC is used to check the quality of the raw (reads) data, generating one HTML report per input Fastq file. Default settings are used with FastQC except for the option 'threads', which should be adapted based on the resources available.

Version: 0.11.8

Input: raw reads Fastq file

Output: One HTML report per input Fastq file

Command (example)

```
> fastqc -o
fastqc_report_output_dir SAMPL1_L001_R1_001.fastq.gz SAMPL1_L001_R2_001.fastq
.gz
```

Raw reads poly A/T tail trimming (Prinseq)

In this step, Prinseq is used to remove poly A/T tails from the 3' or 5' ends of the raw read sequences. Only poly A/T stretches longer than 10 bases are removed.

Version: 0.20.4

Input: paired-end raw reads gzipped Fastq files

Output: fastq file containing read sequences with poly A/T tail removed

Command (example)

```
> gzip -d /path/to/SAMPL1_L001_R1_001.fastq.gz
> prinseq-lite.pl -trim_tail_right 10 -trim_tail_left 10 -fastq
/path/to/SAMPL1_L001_R1_001.fastq -out_good SAMPLE1_1P_prinseq.fastq
```

Note that since Prinseq cannot handle the gzipped file, we need to first unzip the input gzipped fastq file. Please apply the same procedure on *SAMPL1_L001_R2_001.fastq.gz* (reverse reads) to generate *SAMPLE1_2P_prinseq.fastq*.

Reads Quality Trimming (Trimmomatic)

In this step, low quality sequences and adapter sequences are removed from raw read sequences to improve the quality of the data. The tool Trimmomatic is used to perform reads trimming. Expected input consists of paired-end data. Trimmomatic is run in the '**Paired End Mode**', in which it generates four output fastq files: 2 for the 'paired' reads both surviving trimming and 2 for the corresponding 'unpaired' output where only one read from a read pair survived.

Version: 0.38

Input: paired-end raw reads gzipped Fastq files

Output:

- Paired forward reads: *_1P.fq.gz
- Unpaired forward reads: *_1U.fq.gz
- Paired reverse reads: *_2P.fq.gz
- Unpaired forward reads: *_2U.fq.gz

Options

To run Trimmomatic in paired-end mode, invoke the tool with the following command:

- trimmomatic.sh **PE**

The following trimming options are set to perform the quality based trimming:

- **ILLUMINACLIP**:/usr/local/bin/trimmomatic/0.38/adapters/NexteraPE-PE.fa:2:30:10
    - trim NexteraPE adapter sequences (specified in NexteraPE-PE.fa file - if located elsewhere on your system the path above will need to be modified accordingly)
    - allowing 2 mismatches in the seeding phase
    - quality threshold of 30 for the palindromic matches, only the alignments with a quality higher than 30 are retained to trim adapters.
    - quality threshold of 10 for the sequence alignment only matches, only the alignments with a quality higher than 10 are retained to trim adapters.
- **LEADING**:5
    - remove low-quality bases from the beginning of the reads: bases with a Phred score lower than 5 are removed

- **TRAILING**:5
  - remove low-quality bases from the end of the reads: bases with a Phred score lower than 5 are removed
- **SLIDINGWINDOW**:4:10
  - trim the reads with a sliding window of 4 bases and a Phred score cutoff of 10
- **MINLEN**:20
  - minimal length of a trimmed read to be kept (20 bases)

Note that the trimming options must be set in the strict order (from top to bottom) as specified here. As each option invokes a specific trimming module to clean up sequences and the resulting trimmed sequences are used as the input of the next module, a change of the order of the trimming options can potentially result in differently trimmed sequences.

Command (example)

```
> trimmomatic.sh PE -baseout SAMPLE1.fastq.gz -threads 8
/path/to/SAMPLE1_1P_prinseq.fastq  /path/to/SAMPLE1_2P_prinseq.fastq
ILLUMINACLIP:/usr/local/bin/trimmomatic/0.38/adapters/NexteraPE-PE.fa:2:30:10
LEADING:5 TRAILING:5 SLIDINGWINDOW:4:10 MINLEN:20
```

By using the '-baseout' option, it simplifies the output file name specification of Trimmomatic. It will then generate the following output files:

- SAMPLE1_1P.fastq.gz
- SAMPLE1_2P.fastq.gz
- SAMPLE1_1U.fastq.gz
- SAMPLE1_2U.fastq.gz

The unpaired reads are merged together since their origins are not interesting in the downstream analysis. The following command concatenates unpaired forward and reverse reads into one file *SAMPLE1_SE.fastq*. Note that a command line tool '*zcat*' is required for this operation, which should be available or can be easily installed on most of the Linux system.

```
> zcat SAMPLE1_1U.fastq.gz SAMPLE1_2U.fastq.gz > SAMPLE1_SE.fastq
```

Trimmed Reads Quality Check (FastQC)

In this step, FastQC is used to check the quality of the (paired) trimmed reads, generating two HTML reports per input Fastq file. Default settings are used with FastQC except for the option 'threads', which should be adapted based on the resources available.

Version: 0.11.8

Input: raw reads gzipped Fastq files

Output: One HTML report per input Fastq file

Note that only the quality of the paired-end outputs (1P and 2P) of Trimmomatic are checked, as generally, the majority of reads after trimming should remain paired for a good quality NGS run. If this would not be the case, this would be highly suspicious.

Command (example)

```
> fastqc -o fastqc_report_output_dir /path/to/SAMPLE1_1P.fastq.gz
/path/to/SAMPLE1_2P.fastq.gz
```

Reads Taxonomy Profiling (Kraken)

In this step, read sequences are analyzed by Kraken to identify their origin (species as taxonomy id), generating a taxonomic profile of reads.

Version: 1.1

Input: reads gzipped Fastq file(s)

Output: reads taxonomy profiles in tsv format

Options

- --paired: Kraken will run in paired mode to increase sensitivity by analyzing forward and reverse read sequences together
- --threads: set according to the available resources
- --db: the location of the Kraken database

Databases

The Kraken database used in the pipeline is custom built following the guideline described in the Kraken manual. It is created with sequences from the NCBI RefSeq database and contains all 'Complete Genome' sequences for each target taxonomic group and with the accession prefixes NC, NW, AC, NG, NT, NS, and NZ. The genome sequences of the following taxonomic groups are included: archaea, bacteria, fungi, human, protozoa, and viral.

The Kraken database used is generated with genome sequences downloaded from the RefSeq Genome FTP (ftp://ftp.ncbi.nlm.nih.gov/genomes/refseq/) and the taxonomy information downloaded from the NCBI taxonomy FTP (ftp://ftp.ncbi.nih.gov/pub/taxonomy) on **18/02/2019**.

Note that due to the large size of the custom built Kraken database, it can only run on a machine with enough memory to load the complete database (256GB is advised).

The database will be made available by either FTP or programs for sending large files to the METASTAVA partners.

Command (example)

```
> kraken --paired --fastq-input --db /path/to/KRAKEN_DATABASE --output
SAMPLE1_pe_output.tsv --threads 8 /path/to/SAMPLE1_1P.fastq.gz
/path/to/SAMPLE1_2P.fastq.gz
> kraken --fastq-input --db /path/to/KRAKEN_DATABASE --output
SAMPLE1_se_output,tsv --threads 8 /path/to/SAMPLE1_SE.fastq
> cat SAMPLE1_pe_output.tsv SAMPLE1_se_output.tsv >
SAMPLE1_complete_output.tsv
```

This workflow will process both the paired and the unpaired reads separately to prevent loss of information. As demonstrated above, Kraken was applied first on the paired reads, and then on the unpaired ones. The reads profiling results generated by the two Kraken runs are then combined (concatenated) to generate the complete reads taxonomy profiles, *SAMPLE1_complete_output.tsv*. Nevertheless, as mentioned before, a large number of orphaned reads (i.e. single end reads after trimming) should be considered as highly suspicious.

Taxonomy Profile Report Generation (Kraken)

In this step, the 'kraken-report' command from Kraken is used to summarize the read taxonomy profiles generated in the previous step into a report.

Version: 1.1

Input: reads taxonomy profiles generated by Kraken

Output: A summarized report of the reads taxonomy profiles (tsv format)

Databases

Note that the exact same Kraken database used to generate the reads taxonomy profiles should be specified (with the 'db' option) to run 'kraken-report'. Otherwise, the report script might encounter problems and the resulting report will be invalid.

Command (example)

```
> kraken-report --db /path/to/KRAKEN_DATABASE
/path/to/SAMPLE1_complete_output.tsv > SAMPLE1_complete_report.tsv
```

Optional: Taxonomy Profile Visualization (Krona)

In this step, the reads taxonomy profiles generated by Kraken are fed into the Krona tool to generate a self-contained Radial space-filling (RSF) plot allowing interactive data exploration. This step is not necessary but facilitates navigating the output.

Version: 2.7

Input: reads taxonomy profiles generated by Kraken

Output: Krona visualization of taxonomy profile as a HTML page

Options

- --k: Allow assignments to taxa with ranks labeled "no rank" (instead of moving up to parent).
- --tax: Path to directory containing a taxonomy database to use.

Databases

The same NCBI taxonomy database used to build the Kraken database should be provided to Krona in order to properly generate the RSF plot.

Command (example)

```
> cut -f2,3 /path/to/SAMPLE1_complete_output.tsv | ktImportTaxonomy -k -o SAMPLE1_krona.html -tax /path/to/KRONA_DATABASE -
```

Note that in the command above, 'cut' outputs columns 2 and 3 from the kraken reads taxonomy profiles into stdout and the Krona command ktImportTaxonomy takes the data from stdout and generates a RSF visualization in HTML format.

Reference Genome Sequence Indexing (Bowtie2)

In this step, the reference genome sequences are indexed in order to do read mapping using Bowtie2. Note that the most relevant reference sequences of target species should be provided by each participant of the project for their own target species.

Version: 2.3.4.3

Input: reference genome sequence(s) in FASTA format

Output: Bowtie2 indices of genome sequence (multiple files)

Command (example)

```
> bowtie2-build /path/to/REFERENCE.fasta REFERENCE_INDEX_PREFIX
```

Note that this command generates multiple files with 'REFERENCE_INDEX_PREFIX' as basename (file name without extension). In the next read mapping step, only REFERENCE_INDEX_PREFIX needs to be specified to refer to the Bowtie2 genome indices.

Read Mapping Against Target Species (Bowtie2)

In this step, Bowtie2 is used to map trimmed reads on to the reference genome sequence of the target species to identify reads originating from the target species, which will be considered as the Golden Standard to evaluate the performance of the taxonomy profiling tools (e.g., Kraken). The aligned reads contained within the resulting SAM file are considered as the viral content of the investigated species.

Version: 2.3.4.3

Input:

- trimmed reads
- indexed reference genome sequence

Output: SAM file of reads aligned to the reference sequence of target species

Options

- --local
  - Bowtie2 will trim or clip some bases from one or both ends of the alignment to maximize the alignment score in order to identify the best alignment
- --very-sensitive-local
  - preset that produces the most sensitive and accurate alignment by changing the values for several options, albeit has the longest running time
- --phred33
  - the Phred quality encoding used in the Fastq file. The latest one used by Illumina should be Phred33

Command (example)

```
> bowtie2 --local --very-sensitive-local --phred33 -p 8 -x /path/to/REFERENCE_INDEX_PREFIX -1 /path/to/SAMPLE1_1P.fastq.gz -2 /path/to/SAMPLE1_2P.fastq.gz -U /path/to/SAMPLE1_SE.fastq  -S SAMPLE1_mapped.sam
```

Note that the '-p' option specifies the number of threads used by Bowtie2. This needs to be adapted for your system accordingly. All aligned reads in the bam file are considered as part of the target species.

Unique eLabID: 20190307-9768c90715e475f5e595211a72874f1db76b1f31
link : /experiments.php?mode=view&id=176
