# Supplementary material for: An interlaboratory proficiency test using metagenomic sequencing as a diagnostic tool for the detection of RNA viruses in swine fecal material
Source: Microbiol Spectr. 2024 Aug 20;12(10):e04208-23. doi: 10.1128/spectrum.04208-23 (PMC11448438; doi:10.1128/spectrum.04208-23)
Supplement: Supplemental data 2 — Characterization. [file spectrum.04208-23-s0002.pdf]

File S2. characterization of the proficiency test sample

We performed multiple tests of the faecal sample to verify the presence of porcine astrovirus in it and subsequent identification of each species. These included a prior mNGS virome characterization, blast analysis of contigs assembled from PT data, as well as targeted astrovirus real-time RT-PCR assays.

mNGS. Briefly, RNA was extracted using the same approach as described in the methods section, followed by SuperScript IV reverse transcription (Thermo Fisher Scientific/Invitrogen/Life Technologies, Eugene, OR) and NEBnext second strand cDNA synthesis (New England BioLabs, Ipswich, MA). The library was constructed using Nextera XT DNA kit (Illumina, San Diego, CA). DNA quantity and quality were validated using Qubit 2.0 fluorometer (Thermo Fisher Scientific/Invitrogen/Life Technologies, Carlsbad, CA) and Agilent 2100 Bioanalyzer (Agilent Technologies, Waldbronn, Germany). DNA was diluted to 12 pM concentration and sequenced using 600 v3 kit and Illumina MiSeq instrument according to the manufacturer's protocol.

de novo genome assembly was performed using CLC Genomics Workbench v.10.1.1 (Qiagen Bioinformatics, Hilden, Germany), and the assembled contigs were blasted against the NCBI nucleotide database. While porcine astrovirus 1 (PAstV1) is listed as the only official species, additional porcine astroviruses that are phylogenetically distinct from PAstV1, e.g., PAstV2, PAstV3, PAstV4, PAstV5, remain unclassified but are continuously reported in literatures. Furthermore, the NCBI RefSeq Microbial Genomes database lists both classified and unclassified astroviruses as "species". In this study, we followed the same unofficial naming to distinguish those porcine astroviruses. We identified two species of porcine astroviruses. First, PAstV4 was represented by two contigs named 1126 (6197 nt) and 1127 (4770 nt), which were 91.4% identical as determined by NCBI Blast 2 sequences. The contig 1126 was

near complete genome length and most similar to porcine astrovirus 4 (accession number LC201613) with a max score of 4538, E-value of 0, and 83% identity according to NCBI blastn analysis. Secondly, PAstV2 was represented by contigs named 4189 (3033 nt) and 530 (6254 nt) with 80% identity. The contig 530 was most similar to Mamastrovirus 3 (accession number MW504556) with a max score of 3633, E-value of 0, and percentage of identity of 80%. The Blast analyses were performed on 2022-04-24. Due to viral quasispecies and potential multiple introductions, it is possible to have multiple distinguishable sequences representing the same species. Then we aligned sequencing data to the MAstV3 reference sequence NC_025379 by Bowtie2, assembled the mapped contigs by MegaHit (Li et al., 2015), blasted the contigs longer than 300 nucleotides (nt) against the database, and found the contig k119_2 (936 nt) as MAstV3. To gain a better view of the relationship between the contigs and published genomes of porcine astroviruses, we performed phylogenetic analysis of the contigs excluding the short one for PAstV5. We then aligned the five contigs with 70 complete genomes, representing different species of porcine astroviruses, using Seaview 5.0. The porcine astrovirus phylogeny was then inferred using PhyML 3.1 under the GTR + I + G model with 100 bootstrap replicates (Figure S1). The gaps were treated as unknown characters, as default. The phylogenetic analysis confirmed the blasting results that assigned the five contigs into three species, namely, PAstV2, PAstV4 and MAstV3.

As some participants reported two additional species, PastV5 and PAstV3, we mapped the reads from all participants to the reference genome of the above two species and were able to assemble a short contig of PAstV5 (458 nt), but none of PAstV3. Therefore, PAstV5 represented a fourth species.

Astrovirus real-time RT-PCRs. Targeted real-time RT-PCR assays were used to confirm the presence of specific porcine astrovirus species in the test sample. In addition to the PAstV4 real-time RT-PCR assay (Zhou et al., 2016), we developed four new assays for the

specific detection of PAstV2, PAstV5, MAstV3 and PAstV3. AgPath-ID™ One-Step RT-PCR Reagents (ThermoFisher Scientific, Foster City, CA) were used for probe-based detection of the five porcine astrovirus species, following the manufacturer's instructions. A Cq cutoff value was set to 37. As the five assays targeted different genome regions, for each species, we had five synthesized DNA oligos corresponding to the amplicons of the five assays, which resulted in a total of 25 oligos. We then evaluated the specificity of each assay by testing five species-specific pools of oligos at 10^5 copies/reaction, and appropriate controls. We found no cross-reactivity of the assays. The nucleotide sequences of primers and probes for the new assays and synthetic standards (for the target species) or amplicons for non-target species are shown in Table S1. We finally quantified viral load of each species by testing 10-fold dilution serials of the standards, as 5.5×10^5 copies/mg for PAstV2, 9.8×10^4 copies/mg for PAstV4, 2.4×10^3 copies/mg for MAstV3 and 5.6×10^1 copies/mg for PAstV5, and the absence of PAstV3.

References

Zhou W, Ullman K, Chowdry V, Reining M, Benyeda Z, Baule C, Juremalm M, Wallgren P, Schwarz L, Zhou E, Pedrero SP, Hennig-Pauka I, Segales J, Liu L, 2016. Molecular investigations on the prevalence and viral load of enteric viruses in pigs from five European countries. Vet Microbiol 182:75-81.

Bauermann FV, Hause B, Buysse AR, Joshi LR, Diel DG, 2019. Identification and genetic characterization of a porcine hepe-astrovirus (bastrovirus) in the United States. Arch Virol 164:2321-2326.

Li D, Liu CM, Luo R, Sadakane K, Lam TW, 2015. MEGAHIT: an ultra-fast single-node solution for large and complex metagenomics assembly via succinct de Bruijn graph. Bioinformatics 31:1674-1676.
