# Supplementary material for: An interlaboratory proficiency test using metagenomic sequencing as a diagnostic tool for the detection of RNA viruses in swine fecal material
Source: Microbiol Spectr. 2024 Aug 20;12(10):e04208-23. doi: 10.1128/spectrum.04208-23 (PMC11448438; doi:10.1128/spectrum.04208-23)
Supplement: Fig. S1 — Phylogeny. [file spectrum.04208-23-s0003.pdf]

Figure S1. Phylogenetic relationship between the four genome fragments, highlighted in blue, that were assembled from sequencing of the same PT sample prior to the PT, and the astroviruses of porcine and other animal origin. The tree was constructed via PhyML with numbers on branches indicating bootstrap support. The nine porcine astrovirus reference genomes used in Bowtie2 approach are highlighted in red. The bar indicates average changes per site.

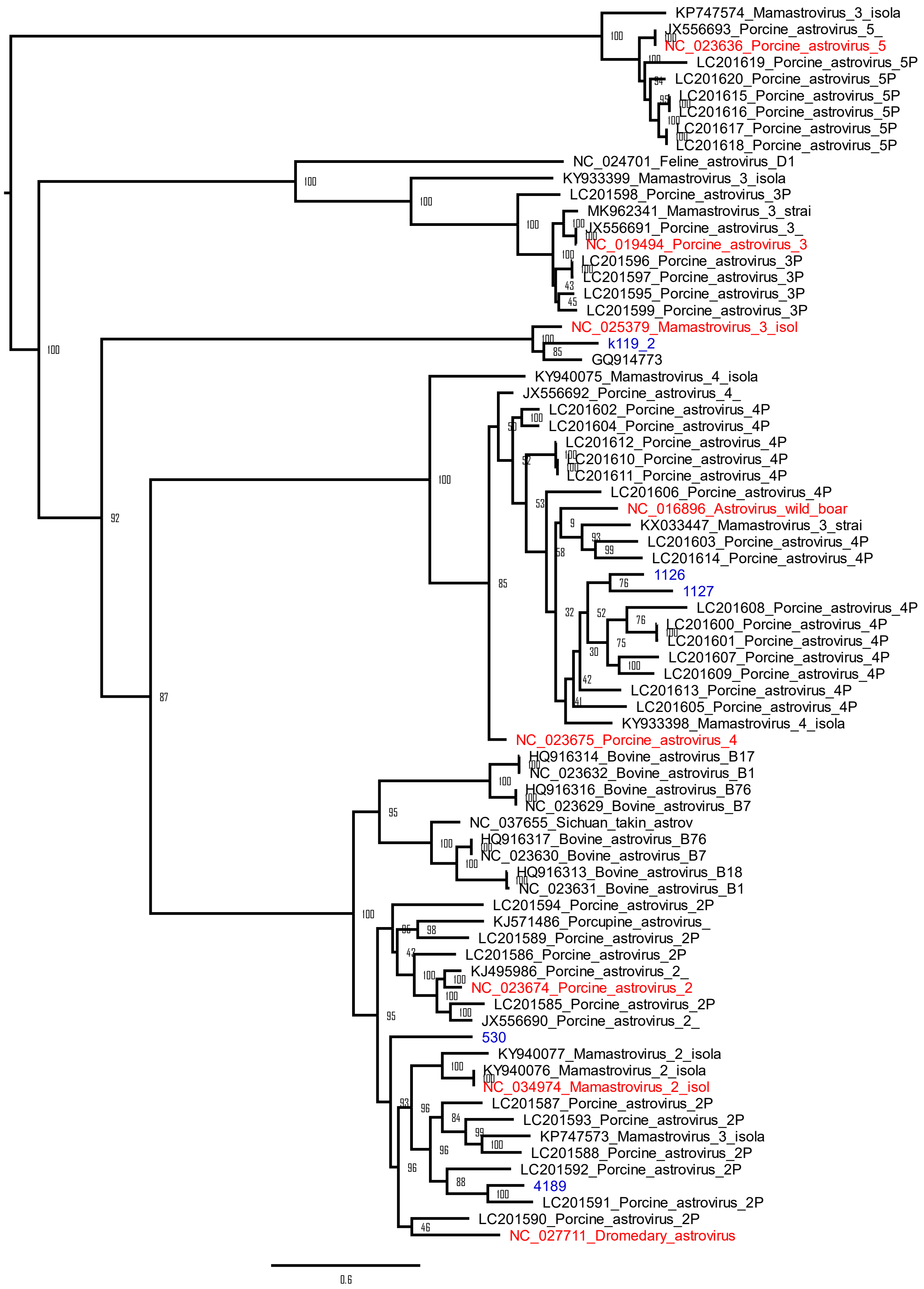
KP747574_Mamastrovirus_3_isola
JX556693_Porcine_astrovirus_5_
NC_023636_Porcine_astrovirus_5
LC201619_Porcine_astrovirus_5P
LC201620_Porcine_astrovirus_5P
LC201615_Porcine_astrovirus_5P
LC201616_Porcine_astrovirus_5P
LC201617_Porcine_astrovirus_5P
LC201618_Porcine_astrovirus_5P
NC_024701_Feline_astrovirus_D1
KY933399_Mamastrovirus_3_isola
LC201598_Porcine_astrovirus_3P
MK962341_Mamastrovirus_3_strai
JX556691_Porcine_astrovirus_3_
NC_019494_Porcine_astrovirus_3
LC201596_Porcine_astrovirus_3P
LC201597_Porcine_astrovirus_3P
LC201595_Porcine_astrovirus_3P
LC201599_Porcine_astrovirus_3P
NC_025379_Mamastrovirus_3_isol
k119_2
GQ914773
KY940075_Mamastrovirus_4_isola
JX556692_Porcine_astrovirus_4_
LC201602_Porcine_astrovirus_4P
LC201604_Porcine_astrovirus_4P
LC201612_Porcine_astrovirus_4P
LC201610_Porcine_astrovirus_4P
LC201611_Porcine_astrovirus_4P
LC201606_Porcine_astrovirus_4P
NC_016896_Astrovirus_wild_boar
KX033447_Mamastrovirus_3_strai
LC201603_Porcine_astrovirus_4P
LC201614_Porcine_astrovirus_4P
1126
1127
LC201608_Porcine_astrovirus_4P
LC201600_Porcine_astrovirus_4P
LC201601_Porcine_astrovirus_4P
LC201607_Porcine_astrovirus_4P
LC201609_Porcine_astrovirus_4P
LC201613_Porcine_astrovirus_4P
LC201605_Porcine_astrovirus_4P
KY933398_Mamastrovirus_4_isola
NC_023675_Porcine_astrovirus_4
HQ916314_Bovine_astrovirus_B17
NC_023632_Bovine_astrovirus_B1
HQ916316_Bovine_astrovirus_B76
NC_023629_Bovine_astrovirus_B7
NC_037655_Sichuan_takin_astrov
HQ916317_Bovine_astrovirus_B76
NC_023630_Bovine_astrovirus_B7
HQ916313_Bovine_astrovirus_B18
NC_023631_Bovine_astrovirus_B1
LC201594_Porcine_astrovirus_2P
KJ571486_Porcupine_astrovirus_
LC201589_Porcine_astrovirus_2P
LC201586_Porcine_astrovirus_2P
KJ495986_Porcine_astrovirus_2_
NC_023674_Porcine_astrovirus_2
LC201585_Porcine_astrovirus_2P
JX556690_Porcine_astrovirus_2_
530
KY940077_Mamastrovirus_2_isola
KY940076_Mamastrovirus_2_isola
NC_034974_Mamastrovirus_2_isol
LC201587_Porcine_astrovirus_2P
LC201593_Porcine_astrovirus_2P
KP747573_Mamastrovirus_3_isola
LC201588_Porcine_astrovirus_2P
LC201592_Porcine_astrovirus_2P
4189
LC201591_Porcine_astrovirus_2P
LC201590_Porcine_astrovirus_2P
NC_027711_Dromedary_astrovirus
0.6
