# Supplementary material for: An interlaboratory proficiency test using metagenomic sequencing as a diagnostic tool for the detection of RNA viruses in swine fecal material
Source: Microbiol Spectr. 2024 Aug 20;12(10):e04208-23. doi: 10.1128/spectrum.04208-23 (PMC11448438; doi:10.1128/spectrum.04208-23)
Supplement: Table S1 — Nucleotide sequences. [file spectrum.04208-23-s0004.pdf]

Table S1. Nucleotide sequences of primers/probes and standards for porcine astrovirus species.

| Name | Sequence* |
|---|---|
| PAstV2-F | CTGTGGGAAACTCCTTAG |
| PAstV2-R | TAGAGCTTGTCTTTGGTC |
| PAstV2-P | 6FAM-TCCCCTCCAAAGACGATCCAG-BHQ1 |
| PAstV3-F | GCACGTAGGCGGAAATGGT |
| PAstV3-R | TCCGTTGCTTGGATTGTTGTT |
| PAstV5-F | TGTGCGCCGTTGTGTTGA |
| PAstV5-R | GATCCTATCTGAAAAGACKGGCA |
| PAstV5-P | 6FAM-TGCGGGCTCGTGTTGGCTCTG-BHQ1 |
| MAstV3-F | AACAACAACTTGACAAATCAGGTGTC |
| MAstV3-R | GTTCCAAGGGTTGCTGTTGC |
| MAstV3-P | 6FAM-CAGGTCCAAAACCAGCAATCCGTCA-BHQ1 |
| PAstV2-LT898434 | AACAGCCCTCTGTGGGAAACTCCTTAGTTACAAGATCTTGATGCACAACAGTGAAGACCATCCATTCAAGGAATACATTGAAAAGTGCCTTGCTGCGCTCGAGGATGGGCAAACCATACCGCGACTCACTGATGAGCAGCTGGATCGTCTTTGGAGGGGAGGACCAAAGACAAGCTCTAATGGCTAACC |
| PAstV3-NC_019494 | aaaaactcagGCACGTAGGCGGAAATGGTATAAGCCCCGTCGACAACAAAATCAGCCCCAACAACAATCCAAGCAACGGActactgtgag |
| PAstV4-GU562296 | CTGATGTTACAGCGCTGCATGGGAAACTCCTGAGCCTACAACTTTTGATGCATAATCATCCTGACAGTGCCTTTAAAGATTACATCAATAAATGTTTGGCTGAAACAGCGAGGCACGCCGAGGATCTGCCTGCAAGACTTACAGAAAGGCAGATGGACAGGCTTTGGAGGGGCGGACCAAAGCATAAGCCTAATGGCTA |
| PAstV5_k119-1 | ttcggcgctaTGTGCGCCGTTGTGTTGATGTCTTGCGGGCTCGTGTTGGCTCTGATTTGCCCGTCTTTTCAGATAGGATCttgtcgtacc |
| MAstV3_k141-7 | aatattgtcaAACAACAACTTGACAAATCAGGTGTCACAGGTCCAAAACCAGCAATCCGTCAACGGGCAACAGCAACCCTTGGAACcattggaagc |

*Probes are labeled with 6FAM at the 5´ end and BHQ1 at the 3´. Extra nucleotides in small letters are added to both ends of standards.
