# Supplementary material for: An interlaboratory proficiency test using metagenomic sequencing as a diagnostic tool for the detection of RNA viruses in swine fecal material
Source: Microbiol Spectr. 2024 Aug 20;12(10):e04208-23. doi: 10.1128/spectrum.04208-23 (PMC11448438; doi:10.1128/spectrum.04208-23)
Supplement: Table S2 — Pairwise sequence identity. [file spectrum.04208-23-s0005.pdf]

Table S2. Pairwise sequence identity of eight astrovirus reference genomes determined after clustalw multiple sequence alignment (BioEdit, version 7.2.5).

Species	Refseq	NC_023675	NC_016896	NC_023674	NC_027711	NC_034974	NC_019494	NC_023636	NC_025379
PAstV4	NC_023675.1	100.0%							
	NC_016896.1	71.3%	100.0%						
PAstV2	NC_023674.1	44.0%	42.9%	100.0%					
	NC_027711.1	43.2%	42.6%	70.1%	100.0%				
	NC_034974.1	44.2%	43.1%	70.5%	69.4%	100.0%			
PAstV3	NC_019494.1	37.6%	36.7%	38.9%	38.7%	40.2%	100.0%		
PAstV5	NC_023636.1	35.5%	35.2%	37.0%	37.1%	37.5%	40.5%	100.0%	
MAstV3	NC_025379.1	41.2%	39.9%	42.4%	42.4%	42.4%	38.8%	36.4%	100.0%
