# Supplementary material for: An interlaboratory proficiency test using metagenomic sequencing as a diagnostic tool for the detection of RNA viruses in swine fecal material
Source: Microbiol Spectr. 2024 Aug 20;12(10):e04208-23. doi: 10.1128/spectrum.04208-23 (PMC11448438; doi:10.1128/spectrum.04208-23)
Supplement: Table S3 — Accession numbers. [file spectrum.04208-23-s0006.pdf]

Table S3. Retrieved sequences and corresponding species

| accession | species | accession | species | accession | species | accession | species |
|---|---|---|---|---|---|---|---|
| AB037272 | MAstV3 | KY047745 | PAstV4 | KJ496038 | PAstV2 | MK378468 | PAstV4 |
| DQ517523 | MAstV3 | KY047746 | PAstV4 | KJ496039 | PAstV2 | MK378469 | PAstV4 |
| GQ174439 | MAstV3 | KY047747 | PAstV4 | KJ496040 | PAstV2 | MK378470 | PAstV5 |
| GQ174440 | MAstV3 | KY047748 | PAstV4 | KJ496041 | PAstV2 | MK378471 | PAstV4 |
| GQ174441 | MAstV3 | KY047749 | PAstV4 | KJ496042 | PAstV2 | MK378472 | PAstV4 |
| GQ174442 | MAstV3 | KY047750 | PAstV4 | KJ496044 | PAstV2 | MK378473 | PAstV4 |
| GQ174443 | MAstV3 | KY047751 | PAstV4 | KJ496045 | PAstV5 | MK378474 | PAstV2 |
| GQ174444 | MAstV3 | KY047752 | PAstV4 | KJ496046 | PAstV2 | MK378475 | PAstV4 |
| GQ174445 | MAstV3 | KY047753 | PAstV4 | KJ496047 | PAstV4 | MK378476 | PAstV4 |
| GQ174446 | MAstV3 | KY047754 | PAstV4 | KJ496048 | PAstV2 | MK378477 | PAstV2 |
| GQ174447 | MAstV3 | KY047755 | PAstV4 | KJ496049 | PAstV2 | MK378478 | PAstV4 |
| GQ174448 | MAstV3 | KY047756 | PAstV4 | KJ496050 | PAstV5 | MK378479 | PAstV2 |
| GQ174449 | MAstV3 | KY047757 | PAstV4 | KJ533341 | PAstV2 | MK378480 | PAstV4 |
| GQ174450 | MAstV3 | KY047758 | PAstV4 | KJ533342 | PAstV2 | MK378481 | PAstV2 |
| GQ174451 | MAstV3 | KY047759 | PAstV4 | KJ533343 | PAstV2 | MK378482 | PAstV4 |
| GQ174452 | MAstV3 | KY047760 | PAstV4 | KJ533344 | MAstV3 | MK378483 | PAstV2 |
| GQ174453 | MAstV3 | KY047761 | PAstV4 | KJ533345 | PAstV2 | MK378484 | PAstV4 |
| GQ174454 | MAstV3 | KY047762 | PAstV4 | KJ533346 | PAstV2 | MK378485 | PAstV4 |
| GQ174455 | MAstV3 | KY073229 | PAstV3 | KJ533347 | PAstV3 | MK378486 | PAstV4 |
| GQ174456 | MAstV3 | KY073230 | PAstV3 | KJ533348 | PAstV2 | MK378487 | PAstV4 |
| GQ174457 | MAstV3 | KY073231 | PAstV3 | KJ533349 | PAstV2 | MK378488 | PAstV4 |
| GQ174458 | MAstV3 | KY073232 | PAstV3 | KJ533350 | PAstV5 | MK378489 | PAstV4 |
| GQ174459 | MAstV3 | KY073233 | PAstV3 | KJ533351 | PAstV2 | MK378490 | PAstV4 |
| GQ174460 | MAstV3 | KY214437 | PAstV4 | KJ533352 | PAstV2 | MK378491 | PAstV4 |
| GQ174461 | MAstV3 | KY214438 | PAstV2 | KJ533353 | PAstV2 | MK378492 | PAstV2 |
| GQ174462 | MAstV3 | KY230626 | PAstV2 | KJ533354 | PAstV2 | MK378493 | PAstV4 |
| GQ174463 | MAstV3 | KY230627 | PAstV2 | KJ533355 | PAstV2 | MK378494 | PAstV4 |
| GQ174464 | MAstV3 | KY230628 | PAstV2 | KJ533356 | PAstV2 | MK378495 | PAstV2 |
| GQ174465 | MAstV3 | KY230629 | PAstV2 | KJ533357 | PAstV2 | MK378496 | PAstV2 |
| GQ174466 | MAstV3 | KY230630 | PAstV2 | KJ533358 | MAstV3 | MK378497 | PAstV2 |
| GQ174467 | MAstV3 | KY230631 | PAstV2 | KJ533359 | PAstV4 | MK378498 | PAstV5 |
| GQ174468 | MAstV3 | KY230632 | PAstV2 | KJ533360 | PAstV5 | MK378499 | PAstV4 |
| GQ174469 | MAstV3 | KY230633 | PAstV2 | KJ533361 | PAstV5 | MK378500 | PAstV4 |
| GQ174470 | MAstV3 | KY230634 | PAstV2 | KJ533362 | PAstV2 | MK378501 | PAstV4 |
| GQ174471 | MAstV3 | KY230635 | PAstV2 | KJ533363 | PAstV2 | MK378502 | PAstV4 |
| GQ174472 | MAstV3 | KY230636 | PAstV2 | KJ533364 | PAstV5 | MK378503 | PAstV5 |
| GQ914773 | MAstV3 | KY230637 | PAstV2 | KJ533365 | PAstV2 | MK378504 | PAstV4 |
| GU562296 | PAstV4 | KY230638 | PAstV2 | KJ533366 | PAstV2 | MK378505 | PAstV2 |
| HQ647381 | PAstV2 | KY230639 | PAstV2 | KJ533367 | PAstV2 | MK378506 | PAstV4 |
| HQ647382 | PAstV2 | KY230640 | PAstV2 | KJ533368 | PAstV2 | MK378507 | PAstV4 |
| HQ647383 | PAstV2 | KY230642 | PAstV2 | KJ533369 | PAstV2 | MK378508 | PAstV4 |
| JF272545 | PAstV4 | KY230643 | PAstV2 | KJ533370 | PAstV2 | MK378509 | PAstV4 |
| JF272546 | PAstV4 | KY230644 | PAstV2 | KJ650561 | PAstV2 | MK378510 | PAstV4 |
| JF272547 | PAstV4 | KY230645 | PAstV2 | KJ650567 | PAstV4 | MK378511 | PAstV4 |
| JF272548 | PAstV4 | KY230646 | PAstV2 | KM083103 | PAstV4 | MK378512 | PAstV4 |
| JF272549 | PAstV4 | KY230647 | PAstV2 | KM083104 | PAstV4 | MK378513 | PAstV5 |
| JF272550 | PAstV4 | KY230648 | PAstV2 | KM211520 | PAstV2 | MK378514 | PAstV4 |
| JF272551 | PAstV4 | KY230649 | PAstV2 | KM211521 | PAstV2 | MK378515 | PAstV4 |

| | | | | | | | |
|---|---|---|---|---|---|---|---|
| JF272552 | PAstV4 | KY230650 | PAstV2 | KM211522 | PAstV2 | MK378516 | PAstV5 |
| JF272553 | PAstV4 | KY230651 | PAstV2 | KM211523 | PAstV4 | MK378517 | PAstV4 |
| JF272554 | PAstV4 | KY230652 | PAstV2 | KM211524 | PAstV2 | MK378518 | PAstV4 |
| JF272555 | PAstV4 | KY230653 | PAstV2 | KM211525 | PAstV2 | MK378519 | PAstV5 |
| JF272556 | PAstV4 | KY412101 | MAstV3 | KM211526 | PAstV5 | MK378520 | PAstV4 |
| JF272557 | PAstV4 | KY412102 | MAstV3 | KM211527 | PAstV2 | MK378521 | PAstV2 |
| JF272558 | PAstV4 | KY412103 | MAstV3 | KM211528 | PAstV2 | MK378522 | PAstV5 |
| JF272559 | PAstV4 | KY412104 | MAstV3 | KM211529 | PAstV2 | MK378523 | PAstV4 |
| JF272560 | PAstV4 | KY412105 | MAstV3 | KP747573 | PAstV2 | MK378524 | PAstV4 |
| JF272561 | PAstV4 | KY412106 | MAstV3 | KP747574 | PAstV5 | MK378525 | PAstV4 |
| JF272562 | PAstV4 | KY412107 | MAstV3 | KP747575 | PAstV5 | MK378526 | PAstV4 |
| JF272563 | PAstV4 | KY412108 | MAstV3 | KP747576 | PAstV5 | MK378527 | PAstV4 |
| JF272564 | PAstV4 | KY412109 | MAstV3 | KP747577 | PAstV5 | MK378528 | PAstV2 |
| JF272565 | PAstV4 | KY412110 | MAstV3 | KP747578 | PAstV5 | MK378529 | PAstV4 |
| JF272566 | PAstV4 | KY412111 | MAstV3 | KP747579 | PAstV5 | MK378530 | PAstV4 |
| JF272567 | PAstV4 | KY412112 | MAstV3 | KP747580 | PAstV5 | MK378531 | PAstV4 |
| JF272568 | PAstV4 | KY412113 | MAstV3 | KP747581 | PAstV5 | MK378532 | PAstV4 |
| JF272569 | PAstV4 | KY412114 | MAstV3 | KP747582 | PAstV5 | MK378534 | PAstV4 |
| JF272570 | PAstV4 | KY412115 | MAstV3 | KP747583 | PAstV5 | MK378535 | PAstV4 |
| JF272571 | PAstV4 | KY412116 | MAstV3 | KP747584 | PAstV5 | MK378536 | PAstV4 |
| JF272572 | PAstV4 | KY412117 | MAstV3 | KP747585 | PAstV5 | MK378537 | PAstV4 |
| JF272573 | PAstV4 | KY412118 | MAstV3 | KP747586 | PAstV5 | MK378538 | PAstV2 |
| JF272574 | PAstV4 | KY412119 | MAstV3 | KP747587 | PAstV5 | MK378539 | PAstV4 |
| JF272575 | PAstV4 | KY412120 | MAstV3 | KP747588 | PAstV5 | MK378540 | PAstV5 |
| JF272576 | PAstV4 | KY412121 | MAstV3 | KP747589 | PAstV2 | MK378541 | PAstV4 |
| JF272577 | PAstV4 | KY412122 | MAstV3 | KP747590 | PAstV2 | MK378542 | PAstV2 |
| JF272578 | PAstV4 | KY412123 | MAstV3 | KP747591 | PAstV2 | MK378543 | PAstV5 |
| JF272579 | PAstV4 | KY412124 | PAstV2 | KP747592 | PAstV2 | MK378544 | PAstV4 |
| JF272580 | PAstV4 | KY412125 | PAstV4 | KP747593 | PAstV2 | MK378545 | PAstV2 |
| JF272581 | PAstV4 | KY412126 | PAstV2 | KP747594 | PAstV2 | MK378546 | PAstV4 |
| JF272582 | PAstV4 | KY412127 | PAstV2 | KP747595 | PAstV2 | MK378547 | PAstV4 |
| JF272583 | PAstV4 | KY412128 | PAstV2 | KP747596 | PAstV2 | MK378548 | PAstV4 |
| JF272584 | PAstV4 | KY412129 | PAstV3 | KP747597 | PAstV2 | MK378549 | PAstV5 |
| JF272585 | PAstV4 | KY412130 | PAstV4 | KP747598 | PAstV2 | MK378550 | PAstV2 |
| JF272586 | PAstV4 | KY412131 | PAstV5 | KP747599 | PAstV2 | MK378551 | PAstV4 |
| JF272587 | PAstV4 | KY412132 | PAstV5 | KP747600 | PAstV2 | MK378552 | PAstV2 |
| JF272588 | PAstV4 | KY412133 | PAstV5 | KP747601 | PAstV2 | MK378553 | PAstV2 |
| JF272589 | PAstV4 | KY412134 | PAstV5 | KP759770 | PAstV2 | MK378554 | PAstV2 |
| JF272590 | PAstV4 | KY412135 | PAstV5 | KP982872 | PAstV2 | MK378555 | PAstV5 |
| JF272591 | PAstV4 | KY412136 | PAstV5 | KT440857 | PAstV5 | MK378556 | PAstV4 |
| JF272592 | PAstV4 | KY412137 | PAstV5 | KT440858 | PAstV2 | MK378557 | PAstV4 |
| JF272593 | PAstV4 | KY412138 | PAstV5 | KT440859 | PAstV2 | MK378558 | PAstV4 |
| JF272594 | PAstV4 | KY412139 | PAstV5 | KT440860 | PAstV5 | MK378559 | PAstV4 |
| JF272595 | PAstV4 | KY412140 | PAstV5 | KT440861 | PAstV5 | MK378560 | PAstV4 |
| JF272596 | PAstV4 | KY412141 | PAstV5 | KT440862 | PAstV2 | MK378561 | PAstV4 |
| JF272597 | PAstV4 | KY412142 | PAstV5 | KT440863 | PAstV2 | MK378562 | PAstV5 |
| JF272598 | PAstV4 | KY940545 | PAstV3 | KT440864 | PAstV2 | MK378564 | PAstV4 |
| JF272599 | PAstV4 | LC201585 | PAstV2 | KT440865 | PAstV2 | MK378565 | PAstV2 |
| JF272600 | PAstV4 | LC201586 | PAstV2 | KT440866 | PAstV2 | MK378566 | PAstV5 |
| JF713710 | PAstV2 | LC201588 | PAstV2 | KT440867 | PAstV2 | MK378567 | PAstV2 |

| | | | | | | | |
|---|---|---|---|---|---|---|---|
| JF713711 | PAstV5 | LC201589 | PAstV2 | KT440868 | PAstV5 | MK378568 | PAstV4 |
| JF713712 | PAstV2 | LC201590 | PAstV2 | KT440869 | PAstV2 | MK378569 | PAstV5 |
| JF713713 | PAstV4 | LC201591 | PAstV2 | KT440870 | PAstV2 | MK378570 | PAstV4 |
| JN088535 | PAstV5 | LC201594 | PAstV2 | KT440871 | PAstV2 | MK378571 | PAstV2 |
| JN088537 | PAstV5 | LC201595 | PAstV3 | KT440872 | PAstV5 | MK378572 | PAstV4 |
| JQ340310 | PAstV4 | LC201596 | PAstV3 | KT440873 | PAstV5 | MK378573 | PAstV4 |
| JQ696831 | PAstV4 | LC201597 | PAstV3 | KT440874 | PAstV5 | MK378574 | PAstV4 |
| JQ696832 | PAstV4 | LC201598 | PAstV3 | KT440875 | PAstV5 | MK378575 | PAstV2 |
| JQ696833 | PAstV4 | LC201599 | PAstV3 | KT440876 | PAstV5 | MK378576 | PAstV2 |
| JQ696834 | PAstV4 | LC201600 | PAstV4 | KT440877 | PAstV5 | MK378577 | PAstV2 |
| JQ696835 | PAstV4 | LC201601 | PAstV4 | KT440878 | PAstV2 | MK378578 | PAstV2 |
| JQ696836 | PAstV4 | LC201603 | PAstV4 | KT757525 | PAstV4 | MK378579 | PAstV2 |
| JQ696837 | PAstV4 | LC201605 | PAstV4 | KT757526 | PAstV4 | MK378580 | PAstV4 |
| JQ696838 | PAstV4 | LC201607 | PAstV4 | KT757527 | PAstV4 | MK378581 | PAstV4 |
| JQ696839 | PAstV4 | LC201608 | PAstV4 | KT757528 | PAstV4 | MK378582 | PAstV2 |
| JQ696840 | PAstV4 | LC201609 | PAstV4 | KT757529 | PAstV4 | MK378583 | PAstV5 |
| JQ696841 | PAstV4 | LC201613 | PAstV4 | KT757530 | PAstV4 | MK378584 | PAstV4 |
| JQ696842 | PAstV4 | LC201614 | PAstV4 | KT757531 | PAstV4 | MK378585 | PAstV4 |
| JQ696843 | PAstV4 | LC201615 | PAstV5 | KT757532 | PAstV4 | MK378586 | PAstV4 |
| JQ696844 | PAstV4 | LC201616 | PAstV5 | KU764484 | PAstV4 | MK378587 | PAstV4 |
| JQ696845 | PAstV4 | LC201617 | PAstV5 | KU764485 | PAstV4 | MK378588 | PAstV2 |
| JQ696846 | PAstV4 | LC201618 | PAstV5 | KU764486 | PAstV4 | MK378589 | PAstV2 |
| JQ696847 | PAstV4 | LC201619 | PAstV5 | KU954449 | PAstV3 | MK378590 | PAstV2 |
| JQ696848 | PAstV4 | LC201620 | PAstV5 | KU954450 | PAstV2 | MK378591 | PAstV4 |
| JQ696849 | PAstV4 | MF138031 | PAstV4 | KU954451 | PAstV5 | MK378592 | PAstV4 |
| JQ696850 | PAstV4 | MF138032 | PAstV4 | KU954452 | PAstV2 | MK460230 | PAstV2 |
| KJ496037 | PAstV2 | MK378467 | PAstV4 | KY047743 | PAstV4 | MW504556 | PAstV2 |
| KY047744 | PAstV4 | MW504552 | PAstV2 | MW504554 | PAstV2 | KJ496036 | PAstV2 |
| JQ696851 | PAstV4 | MF138033 | PAstV4 | KU954453 | PAstV2 | MK460231 | PAstV4 |
| JQ696852 | PAstV4 | MF138034 | PAstV4 | KU954454 | PAstV2 | MK613068 | PAstV4 |
| JQ696853 | PAstV4 | MF138035 | PAstV4 | KU954455 | PAstV2 | MK802129 | PAstV4 |
| JQ696855 | PAstV2 | MF138036 | PAstV4 | KU954456 | PAstV2 | MK802130 | PAstV2 |
| JX232624 | PAstV2 | MF138037 | PAstV4 | KU954458 | PAstV2 | MK802131 | PAstV2 |
| JX232625 | PAstV2 | MF138038 | PAstV4 | KU954461 | PAstV2 | MK802132 | PAstV2 |
| JX232626 | PAstV2 | MF138039 | PAstV4 | KU954462 | PAstV2 | MK802133 | PAstV5 |
| JX232627 | PAstV2 | MF138040 | PAstV4 | KU954463 | PAstV2 | MK802134 | MAstV3 |
| JX519268 | PAstV4 | MF138041 | PAstV4 | KU954464 | PAstV2 | MK802135 | PAstV2 |
| JX519269 | PAstV4 | MF138042 | PAstV4 | KU954466 | PAstV3 | MK802136 | PAstV2 |
| JX519270 | PAstV4 | MF138043 | PAstV4 | KU954467 | PAstV2 | MK802137 | PAstV5 |
| JX519271 | PAstV2 | MF138044 | PAstV2 | KU954468 | PAstV2 | MK802138 | PAstV4 |
| JX519272 | PAstV2 | MF138045 | PAstV4 | KU954469 | PAstV2 | MN136525 | PAstV2 |
| JX519273 | PAstV2 | MF138046 | PAstV2 | KU954470 | PAstV2 | MN136526 | PAstV2 |
| JX519274 | PAstV2 | MF138047 | PAstV4 | KU954472 | PAstV2 | MN136527 | PAstV2 |
| JX519275 | PAstV2 | MF138048 | PAstV4 | KU954473 | PAstV2 | MN136528 | PAstV2 |
| JX519276 | PAstV5 | MF138049 | PAstV4 | KU954474 | PAstV2 | MN136529 | PAstV2 |
| JX519277 | PAstV5 | MF138050 | PAstV4 | KU954475 | PAstV5 | MN136530 | PAstV2 |
| JX519278 | PAstV2 | MF138051 | PAstV4 | KU954476 | PAstV2 | MN136531 | PAstV2 |
| JX519279 | PAstV2 | MF138052 | PAstV4 | KU954477 | PAstV2 | MN136532 | PAstV4 |
| JX519280 | PAstV4 | MF138053 | PAstV4 | KU954478 | PAstV2 | MN136534 | PAstV2 |
| JX519281 | PAstV2 | MF138054 | PAstV4 | KU954479 | PAstV2 | MN136535 | PAstV2 |

| | | | | | | | |
|---|---|---|---|---|---|---|---|
| JX519282 | PAstV2 | MF138055 | PAstV4 | KU954480 | PAstV2 | MN136536 | PAstV2 |
| JX556690 | PAstV2 | MG051040 | PAstV4 | KU954481 | PAstV2 | MT394895 | PAstV3 |
| JX556691 | PAstV3 | MG051041 | PAstV3 | KU954482 | PAstV2 | MT394896 | PAstV3 |
| JX556692 | PAstV4 | MG051042 | PAstV2 | KU954483 | PAstV2 | MT470220 | PAstV4 |
| JX556693 | PAstV5 | MG051043 | PAstV2 | KU954484 | PAstV2 | MT642595 | PAstV5 |
| JX561036 | PAstV4 | MG051044 | PAstV2 | KU954485 | PAstV2 | MW082586 | PAstV2 |
| JX561037 | PAstV4 | MG051045 | PAstV2 | KU954486 | PAstV2 | MW653747 | PAstV3 |
| JX561038 | PAstV4 | MG051046 | PAstV4 | KU954487 | PAstV2 | MW653748 | PAstV3 |
| JX561039 | PAstV4 | MG051047 | PAstV4 | KU954488 | PAstV2 | MW653749 | PAstV3 |
| JX561040 | PAstV4 | MG051048 | PAstV5 | KU954489 | PAstV2 | MW653750 | PAstV3 |
| JX561041 | PAstV4 | MG051049 | PAstV2 | KX033447 | PAstV4 | MW653751 | PAstV3 |
| JX561042 | PAstV4 | MG051050 | PAstV2 | KX060808 | PAstV4 | MW653752 | PAstV3 |
| JX561043 | PAstV4 | MG051051 | PAstV2 | KX060809 | PAstV4 | MW653753 | PAstV3 |
| JX561045 | PAstV4 | MG051052 | PAstV4 | KX431946 | PAstV4 | MW732145 | PAstV3 |
| JX561046 | PAstV4 | MG051053 | PAstV4 | KX431947 | PAstV4 | MW732146 | PAstV3 |
| JX561047 | PAstV4 | MG051054 | PAstV4 | KX431948 | PAstV4 | MW732147 | PAstV3 |
| JX561048 | PAstV4 | MG051055 | PAstV4 | KX431949 | PAstV4 | MW732148 | PAstV3 |
| JX561049 | PAstV4 | MG051056 | PAstV2 | KX431950 | PAstV4 | MW732149 | PAstV3 |
| JX561052 | PAstV4 | MG051057 | PAstV4 | KX453785 | PAstV4 | MW732150 | PAstV3 |
| JX561053 | PAstV4 | MG051058 | MAstV3 | KX453786 | PAstV4 | MW732151 | PAstV3 |
| JX561055 | PAstV4 | MG051059 | PAstV2 | KX453787 | PAstV4 | MW732152 | PAstV3 |
| JX561057 | PAstV4 | MG051060 | PAstV2 | KX453788 | PAstV4 | MW732153 | PAstV3 |
| JX561058 | PAstV4 | MG051061 | PAstV2 | KX453789 | PAstV4 | MW732154 | PAstV3 |
| JX561059 | PAstV4 | MG051062 | PAstV2 | KX453790 | PAstV4 | MW732155 | PAstV3 |
| JX561062 | PAstV4 | MG051063 | PAstV4 | KX453791 | PAstV4 | MW732156 | PAstV3 |
| JX561063 | PAstV4 | MG051064 | PAstV4 | KX453792 | PAstV4 | MW732157 | PAstV3 |
| JX561064 | PAstV4 | MG930777 | PAstV2 | KY047664 | PAstV5 | MW962975 | PAstV4 |
| JX561065 | PAstV4 | MH064173 | PAstV5 | KY047665 | PAstV5 | MZ515542 | PAstV4 |
| JX561066 | PAstV4 | MH064174 | PAstV5 | KY047666 | PAstV5 | MZ515543 | PAstV4 |
| JX561067 | PAstV4 | MH064175 | PAstV5 | KY047667 | PAstV5 | MZ515544 | PAstV4 |
| JX561068 | PAstV4 | MH064176 | PAstV5 | KY047668 | PAstV5 | MZ515545 | PAstV4 |
| JX561070 | PAstV4 | MH399893 | PAstV2 | KY047669 | PAstV5 | MZ515546 | PAstV4 |
| JX561071 | PAstV4 | MH399894 | PAstV2 | KY047670 | PAstV5 | MZ515548 | PAstV4 |
| JX561073 | PAstV4 | MH399895 | PAstV2 | KY047671 | PAstV5 | MZ515550 | PAstV4 |
| JX561076 | PAstV4 | MH399896 | PAstV2 | KY047672 | PAstV5 | NC_016896 | PAstV4 |
| JX561077 | PAstV4 | MH399897 | PAstV2 | KY047673 | PAstV5 | NC_019494 | PAstV3 |
| JX561079 | PAstV4 | MH399898 | PAstV2 | KY047674 | PAstV5 | NC_023636 | PAstV5 |
| JX561081 | PAstV4 | MH399899 | PAstV2 | KY047675 | PAstV5 | NC_023674 | PAstV2 |
| JX561082 | PAstV4 | MH399900 | PAstV5 | KY047676 | PAstV5 | NC_023675 | PAstV4 |
| JX561083 | PAstV4 | MH399901 | PAstV2 | KY047677 | PAstV5 | NC_025379 | MAstV3 |
| JX561084 | PAstV4 | MH399902 | PAstV2 | KY047678 | PAstV5 | JQ696856 | PAstV2 |
| JX561085 | PAstV4 | MH399903 | PAstV2 | KY047679 | PAstV5 | JQ696854 | PAstV2 |
| JX561086 | PAstV4 | MH399904 | PAstV2 | KY047680 | PAstV5 | KJ496043 | PAstV4 |
| JX561088 | PAstV4 | MH399905 | PAstV2 | KY047681 | PAstV5 | JX561087 | PAstV4 |
| JX561089 | PAstV4 | MH399906 | PAstV2 | KY047682 | PAstV5 | JX561080 | PAstV4 |
| JX561090 | PAstV4 | MH399907 | PAstV4 | KY047683 | PAstV5 | JX561078 | PAstV4 |
| JX684071 | PAstV4 | MH399908 | PAstV2 | KY047684 | PAstV5 | JX561075 | PAstV4 |
| JX684072 | PAstV5 | MH399909 | PAstV2 | KY047685 | PAstV5 | JX561074 | PAstV4 |
| KC790414 | PAstV2 | MH399910 | PAstV2 | KY047686 | PAstV5 | JX561072 | PAstV4 |
| KC790415 | PAstV5 | MH399911 | PAstV5 | KY047687 | PAstV5 | JX561069 | PAstV4 |

| | | | | | | | |
|---|---|---|---|---|---|---|---|
| KC790416 | PAstV5 | MH414519 | PAstV2 | KY047688 | PAstV5 | JX561061 | PAstV4 |
| KC790417 | PAstV5 | MH414520 | PAstV2 | KY047689 | PAstV5 | JX561060 | PAstV4 |
| KC790418 | PAstV5 | MH414521 | PAstV2 | KY047690 | PAstV5 | JX561056 | PAstV4 |
| KF211427 | MAstV3 | MH414522 | PAstV2 | KY047691 | PAstV5 | JX561054 | PAstV4 |
| KF211428 | MAstV3 | MH414523 | PAstV4 | KY047692 | PAstV5 | JX561051 | PAstV4 |
| KF211429 | MAstV3 | MH414524 | PAstV4 | KY047693 | PAstV5 | JX561050 | PAstV4 |
| KF787112 | MAstV3 | MH414525 | PAstV4 | KY047694 | PAstV5 | JX561044 | PAstV4 |
| KJ495986 | PAstV2 | MH425243 | PAstV4 | KY047695 | PAstV5 | KY230641 | PAstV2 |
| KJ495987 | PAstV2 | MH511524 | PAstV2 | KY047696 | PAstV5 | KY047718 | MAstV3 |
| KJ495988 | PAstV2 | MH511525 | PAstV4 | KY047697 | PAstV5 | JN088536 | PAstV5 |
| KJ495989 | PAstV2 | MH511526 | PAstV2 | KY047698 | PAstV5 | JN088534 | PAstV5 |
| KJ495990 | PAstV2 | MH511529 | PAstV2 | KY047699 | PAstV5 | MZ515549 | PAstV4 |
| KJ495991 | PAstV3 | MH511530 | PAstV2 | KY047700 | PAstV5 | MZ515547 | PAstV4 |
| KJ495992 | PAstV2 | MH511532 | PAstV2 | KY047701 | PAstV5 | MZ515541 | PAstV4 |
| KJ495993 | PAstV4 | MH511533 | PAstV2 | KY047702 | PAstV5 | LC201612 | PAstV4 |
| KJ495994 | PAstV3 | MH511534 | PAstV2 | KY047703 | MAstV3 | LC201611 | PAstV4 |
| KJ495995 | PAstV4 | MH511535 | PAstV4 | KY047704 | MAstV3 | LC201610 | PAstV4 |
| KJ495996 | PAstV5 | MH511536 | PAstV2 | KY047705 | MAstV3 | LC201606 | PAstV4 |
| KJ495997 | PAstV4 | MH686391 | PAstV3 | KY047706 | MAstV3 | LC201604 | PAstV4 |
| KJ495998 | PAstV3 | MH686392 | PAstV3 | KY047707 | MAstV3 | LC201602 | PAstV4 |
| KJ495999 | PAstV3 | MH686393 | PAstV3 | KY047708 | MAstV3 | LC201593 | PAstV2 |
| KJ496000 | PAstV2 | MH686394 | PAstV3 | KY047709 | MAstV3 | LC201592 | PAstV2 |
| KJ496001 | PAstV2 | MH686395 | PAstV3 | KY047710 | MAstV3 | LC201587 | PAstV2 |
| KJ496004 | MAstV3 | MH686396 | PAstV3 | KY047711 | MAstV3 | KJ496005 | PAstV2 |
| KJ496006 | PAstV2 | MH686397 | PAstV3 | KY047712 | MAstV3 | KJ496003 | PAstV2 |
| KJ496007 | PAstV2 | MH686398 | PAstV3 | KY047713 | MAstV3 | KJ496002 | PAstV2 |
| KJ496008 | PAstV2 | MH686399 | PAstV3 | KY047714 | MAstV3 | KU954465 | PAstV5 |
| KJ496009 | PAstV2 | MH686400 | PAstV3 | KY047715 | MAstV3 | MK378563 | PAstV4 |
| KJ496010 | PAstV2 | MK378441 | PAstV2 | KY047716 | MAstV3 | MK378533 | PAstV4 |
| KJ496011 | PAstV5 | MK378442 | PAstV2 | KY047717 | MAstV3 | MK378440 | PAstV2 |
| KJ496012 | PAstV5 | MK378443 | PAstV2 | KY047719 | PAstV2 | MN136533 | MAstV3 |
| KJ496013 | PAstV2 | MK378444 | PAstV4 | KY047720 | PAstV2 | KU954471 | MAstV3 |
| KJ496014 | PAstV2 | MK378445 | PAstV4 | KY047721 | PAstV2 | KU954460 | MAstV3 |
| KJ496015 | PAstV5 | MK378446 | PAstV4 | KY047722 | PAstV2 | KU954459 | MAstV3 |
| KJ496016 | PAstV2 | MK378447 | PAstV2 | KY047723 | PAstV2 | KU954457 | MAstV3 |
| KJ496017 | PAstV5 | MK378448 | PAstV4 | KY047724 | PAstV2 | MW504546 | MAstV3 |
| KJ496018 | PAstV2 | MK378449 | PAstV4 | KY047725 | PAstV2 | Y15938 | MAstV3 |
| KJ496019 | PAstV5 | MK378450 | PAstV2 | KY047726 | PAstV2 | HM756258 | MAstV3 |
| KJ496020 | PAstV2 | MK378451 | PAstV2 | KY047727 | PAstV2 | KJ571486 | PAstV2 |
| KJ496021 | PAstV2 | MK378452 | PAstV2 | KY047728 | PAstV2 | MW504565 | PAstV2 |
| KJ496022 | PAstV5 | MK378453 | PAstV4 | KY047729 | PAstV2 | KY940076 | PAstV2 |
| KJ496023 | PAstV2 | MK378454 | PAstV4 | KY047730 | PAstV2 | NC_034974 | PAstV2 |
| KJ496024 | PAstV4 | MK378455 | PAstV4 | KY047731 | PAstV4 | KY940077 | PAstV2 |
| KJ496025 | PAstV2 | MK378456 | PAstV4 | KY047732 | PAstV4 | LT898434 | PAstV2 |
| KJ496026 | PAstV5 | MK378457 | PAstV4 | KY047733 | PAstV4 | MW504547 | PAstV2 |
| KJ496027 | PAstV5 | MK378458 | PAstV4 | KY047734 | PAstV4 | MK962341 | PAstV3 |
| KJ496028 | PAstV4 | MK378459 | PAstV4 | KY047735 | PAstV4 | KY933399 | PAstV3 |
| KJ496029 | PAstV2 | MK378460 | PAstV2 | KY047736 | PAstV4 | KY940075 | PAstV4 |
| KJ496030 | PAstV2 | MK378461 | PAstV2 | KY047737 | PAstV4 | MW504549 | PAstV4 |
| KJ496031 | PAstV2 | MK378462 | PAstV2 | KY047738 | PAstV4 | KY933398 | PAstV4 |

| | | | | | | | |
|---|---|---|---|---|---|---|---|
| KJ496032 | PAstV2 | MK378463 | PAstV2 | KY047739 | PAstV4 | MT642666 | PAstV4 |
| KJ496033 | PAstV2 | MK378464 | PAstV4 | KY047740 | PAstV4 | MW504572 | PAstV4 |
| KJ496034 | PAstV2 | MK378465 | PAstV4 | KY047741 | PAstV4 | MW504571 | PAstV4 |
| MK378466 | PAstV4 | KY047742 | PAstV4 | MW504555 | PAstV2 | | |
